# Epithelial-mesenchymal transition classification of circulating tumor cells predicts clinical outcomes in progressive nasopharyngeal carcinoma

**DOI:** 10.3389/fonc.2022.988458

**Published:** 2022-09-21

**Authors:** Jiazhang Wei, Weiming Deng, Jingjin Weng, Min Li, Guiping Lan, Xiang Li, Linsong Ye, Yongli Wang, Fei Liu, Huashuang Ou, Yunzhong Wei, Wenlin Huang, Sifang Xie, Guohu Dong, Shenhong Qu

**Affiliations:** ^1^ Department of Otolaryngology & Head and Neck, The People’s Hospital of Guangxi Zhuang Autonomous Region, Nanning, China; ^2^ Institute of Oncology, Guangxi Academy of Medical Sciences, Nanning, China; ^3^ Key Laboratory of Early Prevention and Treatment for Regional High Frequency Tumor, Ministry of Education/Guangxi Key Laboratory of Early Prevention and Treatment for Regional High Frequency Tumor, Nanning, China; ^4^ Research Center of Medical Sciences, The People’s Hospital of Guangxi Zhuang Autonomous Region, Nanning, China

**Keywords:** nasopharyngeal carcinoma, circulating tumor cells, metastasis, prognosis, epithelial-mesenchymal transition

## Abstract

**Background:**

Liquid biopsy facilitates the enrichment and isolation of circulating tumor cells (CTCs) in various human cancers, including nasopharyngeal carcinoma (NPC). Characterizing CTCs allows observation of the evolutionary process of single tumor cells undergoing blood-borne dissemination, such as epithelial-mesenchymal transition. However, the prognostic value of phenotypic classification of CTCs in predicting the clinical outcomes of NPC remains poorly understood.

**Patients and methods:**

A total of 92 patients who met the inclusion criteria were enrolled in the present study. The CanPatrol™ CTC technology platform was employed to isolate CTCs, and an RNA *in situ* hybridization-based system was used for phenotypic classification. Kaplan–Meier survival curves were used for univariate survival analysis, and the log-rank test was performed for between-group comparisons of the survival curves.

**Results:**

CTCs were detected in 88.0% (81/92) of the enrolled patients with NPC. The total CTC number did not vary between the T and N stages or between Epstein–Barr virus DNA-positive and -negative cases. The numbers of total CTCs and epithelial/mesenchymal (E/M) hybrid CTCs decreased significantly at 3 months post concurrent chemoradiotherapy (*P*=0.008 and *P*=0.023, respectively), whereas the numbers of epithelial or mesenchymal CTCs did not decrease. E/M hybrid-predominant cases had lower disease-free survival (*P*=0.043) and distant metastasis-free survival (*P*=0.046) rates than non-E/M hybrid-predominant cases.

**Conclusion:**

CTC classification enables a better understanding of the cellular phenotypic alterations responsible for locoregional invasion and distant metastasis in NPC. E/M hybrid-predominant CTC distribution predicts unfavorable clinical outcomes in patients with progressive NPC.

## Introduction

Nasopharyngeal carcinoma (NPC) is a highly invasive Epstein–Barr virus (EBV)-associated malignancy with an uneven distribution across races and geographic regions (1, 2). NPC is prevalent in southern China, where an extremely high incidence has been reported in the Cantonese-speaking population (3, 4). NPC at a non-progressive stage is generally well-controlled, which is mainly ascribed to its therapeutic responsiveness to both radiotherapy and chemotherapy. In contrast, progressive NPC often shows a strong tendency to metastasize to the cervical lymph nodes and secondary organs (5). Metastatic disease indicates treatment failure as well as poor prognosis in NPC. Remote metastasis remains the primary cause of cancer-associated mortality in patients with NPC (5, 6).

Circulating tumor cells (CTCs) represent a class of malignant cells derived from primary tumors that successfully infiltrate the blood circulation and are capable of forming metastatic clones at distant sites (7). The recently emerged liquid biopsy technology has enabled surveillance of the evolutionary process during bloodstream metastasis at the single-cell level (8, 9). The capture of CTCs enables the characterization of metastatic tumor cells and advances our understanding of the tumor invasion–metastasis cascade (10). Lin et al. first reported the capture of CTCs in peripheral venous blood by identifying CK-19 positive cells in NPC (11). Previous studies by our group and others have shown that CTCs can be detected in patients with NPC at all stages (12, 13). The expression levels of specific biomolecules in CTCs, such as human telomerase reverse transcriptase, cyclooxygenase-2, and fibronectin 1, have been reported to be associated with NPC prognosis (14–17). Furthermore, Luo et al. indicated that blood-borne disseminated NPC cells exhibit epithelial-mesenchymal transition (EMT) and cancer stem cell (CSC) characteristics, which are closely associated with their metastatic potential (18). Previously, we employed an RNA *in situ* hybridization (RNA ISH)-based platform to classify CTC subpopulations according to phenotypic characteristics (12). Of the epithelial, mesenchymal, and epithelial/mesenchymal (E/M) hybrid CTCs, we found that the E/M hybrid CTCs were closely associated with EBV infection, with the highest levels of EBV DNA, EBV-specific viral capsid antigen (VCA)-IgA, and early antigen (EA)-IgA detected in this CTC subpopulation (12). In a later study, we further investigated distinct mutational signatures among primary tumors and CTCs using whole-exome sequencing (19). However, the significance of the distribution of E/M hybrid CTCs in predicting prognosis in patients with NPC, especially in evaluating the risk of locoregional and metastatic relapses after standard first-line treatment, remains poorly understood. In this follow-up study, we investigated the clinical outcomes in a cohort of patients with NPC with diverse distributions of CTC subpopulations based on phenotypic classification. Our results indicated that the E/M hybrid-predominant distribution of CTCs predicts an unfavorable prognosis in patients with progressive NPC.

## Materials and methods

### Study design and patient enrollment

Patients pathologically diagnosed with NPC between November 2014 and November 2015 at the People’s Hospital of Guangxi Zhuang Autonomous Region were recruited. Patients who met the following inclusion criteria were enrolled: (1) histopathological diagnosis based on nasopharyngeal biopsy samples, (2) Karnofsky score ≥80, (3) completed standard concurrent chemoradiotherapy (CCRT), and (4) provision of written informed consent. The exclusion criteria were as follows: (1) distant metastases established before treatment; (2) intolerance to CCRT; (3) severely impaired liver or kidney function, bleeding disorders, diabetes, or other systemic diseases that could potentially affect the treatment; (4) premature treatment discontinuation; and (5) refusal to undergo the CTC test. For each patient, staging was performed according to the seventh edition of the Union for International Cancer Control staging guidelines. All patients recruited for this study provided written informed consent for the use of their clinical data in non-commercial medical research. The study adhered to the ethical principles of the Declaration of Helsinki. The study protocol was approved by the Ethics Committee of the People’s Hospital of Guangxi Zhuang Autonomous Region (ethical application reference no. Keyan-NSFC-2017-23). The patient enrollment procedure is summarized in the **Supplementary Materials (Figure S1)**.

### CCRT

The standard first-line treatment regimens for all enrolled patients with NPC have been previously described (20, 21). For intensity-modulated radiation therapy, a prescribed dose of 69–72 Gy was delivered to nasopharyngeal lesions (gross tumor volume of the primary nasopharyngeal tumor) and cervical lesions (gross tumor volume of cervical lymph node lesions). A prescribed dose of 60–65 Gy was delivered to the clinical target volume, which included the sites of tumor invasion and lymphatic drainage area. A prescribed dose of 50–56 Gy was delivered to the low-risk lymphatic drainage area. Level IV and V_B_ areas were not irradiated in patients with N_0_ stage disease. The following regimens were used for chemotherapy: (1) cisplatin (30 g/m^2^, intravenous, days 1–3) and 5-fluorouracil (5-FU) (2000 mg/m^2^, continuous intravenous drip for 120 h), every 28 days for two courses, and (2) nedaplatin (80 mg/m^2^, intravenous, day 1) and 5-FU (2000 mg/m^2^, continuous intravenous drip for 120 h), every 28 days for two courses.

### Isolation and RNA ISH-based classification of CTCs

The methodology used for the capture and enrichment of CTCs from peripheral blood and the classification of CTCs by identifying epithelial-to-mesenchymal transition markers was established by Wu et al. (22, 23). The protocol used in the present study was optimized with slight modifications for CTC detection in NPC as described in our previous study (12). Briefly, 5 mL of peripheral blood was collected from patients and stored in anticoagulant tubes for CTC detection within 4 h. The CanPatrol™ CTC enrichment system was used to efficiently isolate CTCs by size as previously described (12, 16, 19). Red blood cell lysis buffer (154 mmol/L NH_4_Cl, 10 mmol/L KHCO_3_, and 0.1 mmol/L EDTA) was administered to eliminate erythrocytes in blood samples. The remaining cells were resuspended in phosphate-buffered saline containing 4% formaldehyde for 5 min. The cells were then filtered using a calibrated membrane with 8-μm-diameter pores (Millipore, Billerica, MA, USA) under at least 0.08 MPa using a filtration system (SurExam, Guangzhou, China), as previously described (12, 16).

RNA ISH was performed to detect the target sequence encoding the molecule of interest, using branched deoxyribonucleic acid (bDNA) signal amplification technology. The cells were pretreated with protease before hybridization with the indicated capture probe specific for EpCAM and CK8/18/19 (epithelial biomarkers), vimentin and twist (mesenchymal biomarkers), and CD45 (leukocyte biomarker). The capture probe sequences were the same as those described in previous studies (12, 16). The cells were washed using a wash buffer [0.1× saline sodium citrate (SSC); Sigma-Aldrich, St. Louis, MO, USA] to remove unbound probes after incubation at 42°C for 2 h. For signal amplification, the cells were incubated with 100 μL preamplifier solution (30% horse serum, 1.5% sodium dodecyl sulfate, 3 mmol/L Tris-HCl [pH 8.0], and 0.5 fmol of the preamplifier) at 42°C for 2 h. The membranes were washed three times with wash buffer (0.1× SSC), followed by incubation with 100 μL of amplifier solution (30% horse serum, 1.5% sodium dodecyl sulfate, 3 mM Tris-HCl [pH 8.0], and 1 fmol of the amplifier). The sequences used for the bDNA signal amplification probes have been previously reported (12, 16). Probes conjugated with the fluorescent dyes Alexa Fluor 594 (for EpCAM and CK8/18/19), Alexa Fluor 488 (for vimentin and twist), and Alexa Fluor 647 (for CD45) were administered, followed by incubation at 42°C for 20 min. After washing with 0.1× SSC, cells were stained with DAPI (Sigma-Aldrich, St. Louis, MO, USA) and observed under a fluorescence microscope with a 100× oil objective (BX53; Olympus, Tokyo, Japan). CTC detection was performed at three predesignated time points: before CCRT, at the end of CCRT, and 3 months after CCRT.

### Follow-up

After discharge from the hospital post-CCRT, all patients were required to be hospitalized for follow-up review every 3 months during the first year, every 4–6 months during the second to third years, and every 12 months after the third year. The following examinations were performed at each follow-up visit: (1) contrast-enhanced magnetic resonance imaging of the nasopharynx, skull base, and cervical lymph nodes; (2) chest computed tomography; (3) high-definition endoscopic examination through both the white-light and narrow-band imaging modes, as described in our previous study (24); (4) ultrasound of the entire abdomen; and (5) if necessary, ^18^F-FDG positron emission tomography integrated with computed tomography to confirm suspected local tumor recurrence or distant relapse that could not be diagnosed by biopsy.

### Endpoints

The endpoints were overall survival (OS), disease-free survival (DFS), local relapse-free survival (LRFS), and distant metastasis-free survival (DMFS). OS was defined as the time from NPC diagnosis to death from any cause, or the date of the last follow-up. DFS was defined as the time from diagnosis to death, disease progression (locoregional recurrence or distant metastasis), or the date of the last follow-up. LRFS was defined as the time from diagnosis to the date of the first recurrence (locoregional) or the last follow-up. DMFS was defined as the time from diagnosis to the date of first distant metastasis or death from any cause.

### Statistical analysis

Data are presented as median (range) or mean ± standard deviation, unless otherwise stated. Data were analyzed using SPSS software (version 19.0; IBM, Chicago, IL, USA). Kruskal–Wallis one-way analysis of variance by rank was used to compare CTC numbers among different T and N stages. The Mann–Whitney U test was used to compare CTC numbers between the EBV DNA-positive and -negative groups. Pearson’s correlation coefficient was calculated to evaluate the correlation between CTC number and tumor size. The unpaired Student’s *t*-test was used to compare EBV DNA levels between groups, while chi-square or Fisher’s exact test was used to determine the differences between the groups. The paired-sample Wilcoxon test was used to analyze changes in CTC numbers detected at the three designated time points. Kaplan–Meier survival curves were used for univariate survival analysis. The log-rank test was performed to compare survival curves between the groups. Statistical significance was set at *P*<0.05.

## Results

### Correlation between total CTC number and clinical characteristics

**Figure 1:**
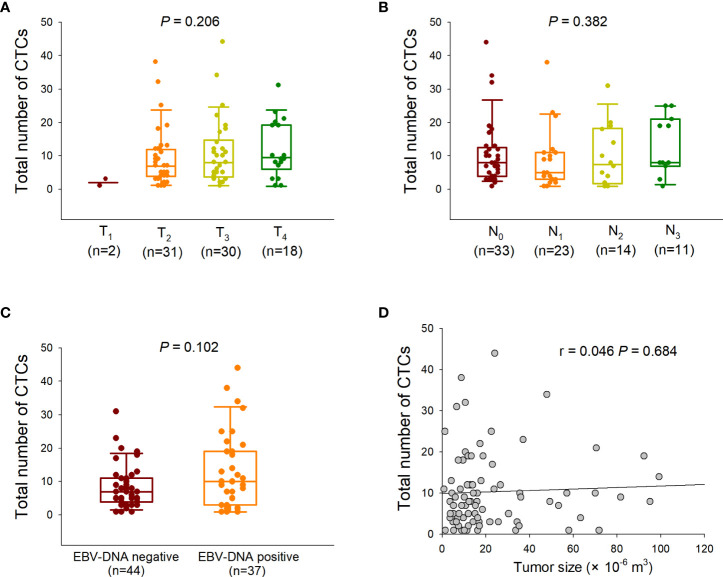
Correlation between total circulating tumor cell (CTC) numbers and clinical characteristics. **(A)** Comparison of the total CTC numbers among different T stages (Kruskal–Wallis one-way analysis of variance). The CTC counts are presented as vertical boxes with error bars indicating the median, 10^th^, 25^th^, 75^th^, and 90^th^ percentiles. **(B)** Comparison of the total CTC numbers among different N stages (Kruskal–Wallis one-way analysis of variance). **(C)** Comparison of the total CTC numbers between Epstein–Barr virus (EBV) DNA-negative and -positive groups (Mann–Whitney U test). **(D)** Correlation between the total CTC numbers and tumor sizes (Pearson correlation analysis).

Before treatment, CTCs were detected in 88.0% (81/92) of the 92 NPC patients enrolled in the present study. A total of 838 CTCs were detected in 81 patients, and the median CTC number was eight (range, 1–44). We evaluated the differences in CTC numbers among the different T and N stages. The differences in CTC numbers across the T and N stages were not significant (**Figures 1A, B**). Furthermore, no difference in the number of CTCs was observed between the EBV DNA-positive and -negative groups (**Figure 1C**). In addition, the total CTC count was not positively correlated with tumor size (**Figure 1D**).

### Phenotypic classification of CTCs using RNA ISH

**Figure 2:**
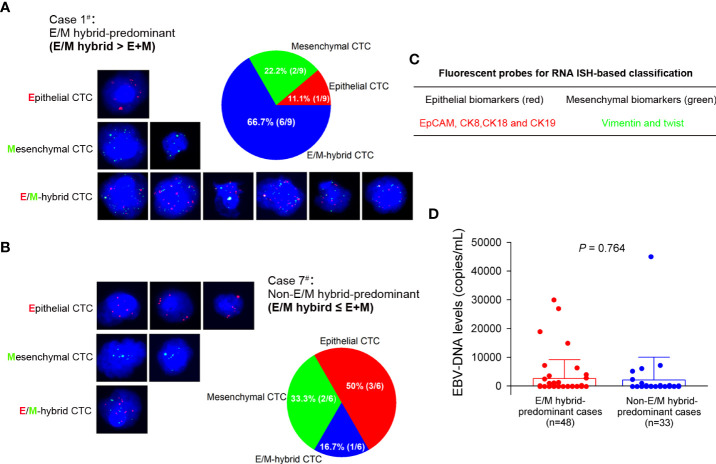
RNA *in situ* hybridization-based phenotypic classification of circulating tumor cells (CTCs) and plasma Epstein–Barr virus (EBV) DNA level in each group. **(A)** A representative phenotypic classification of CTCs from a patient (case 1^#^) with nasopharyngeal carcinoma is shown. Cells stained with epithelial (red fluorescent probe) and mesenchymal (green fluorescent probe) markers were classified as epithelial and mesenchymal CTCs, respectively. Cells stained with both red and green fluorescent probes were classified as epithelial/mesenchymal (E/M) hybrid CTCs. Nine CTCs were detected in case 1, of which 66.7% (6/9) were E/M hybrid CTCs. The patient was assigned to the E/M hybrid-predominant group for follow-up. **(B)** Six CTCs were detected in another patient (case 7^#^), of which 16.7% (1/6) were E/M hybrid CTCs. The patient was assigned to the non-E/M hybrid-predominant group. The cells were analyzed under a fluorescence microscope with a 100× oil objective. **(C)** The various epithelial and mesenchymal makers are indicated with red and green fluorescent probes, respectively. **(D)** Comparison of the plasma EBV DNA level between the E/M hybrid-predominant and non-E/M hybrid-predominant groups.

CTCs were classified into three phenotypes based on simultaneous detection of fluorescence-tagged epithelial and mesenchymal markers. Cells labeled solely by red fluorescence were defined as epithelial CTCs, whereas cells labeled only by green fluorescence were defined as mesenchymal CTCs (**Figures 2A–C**). Cells with both green and red fluorescence were defined as E/M hybrid CTCs (**Figures 2A–C**). A purple fluorescent probe that labels CD45, a classic leukocyte marker, was used to remove leukocytes, as described in our previous study (11). Representative fluorescence microscopic images for phenotypic classification and distribution of each CTC subpopulation are shown in **Figures 2A–C**. In addition, plasma EBV DNA levels detected in the E/M hybrid-predominant group did not differ significantly from those in the non-E/M hybrid-predominant group (**Figure 2D**).

### Changes in CTC numbers and phenotypic distributions before and after treatment

**Figure 3:**
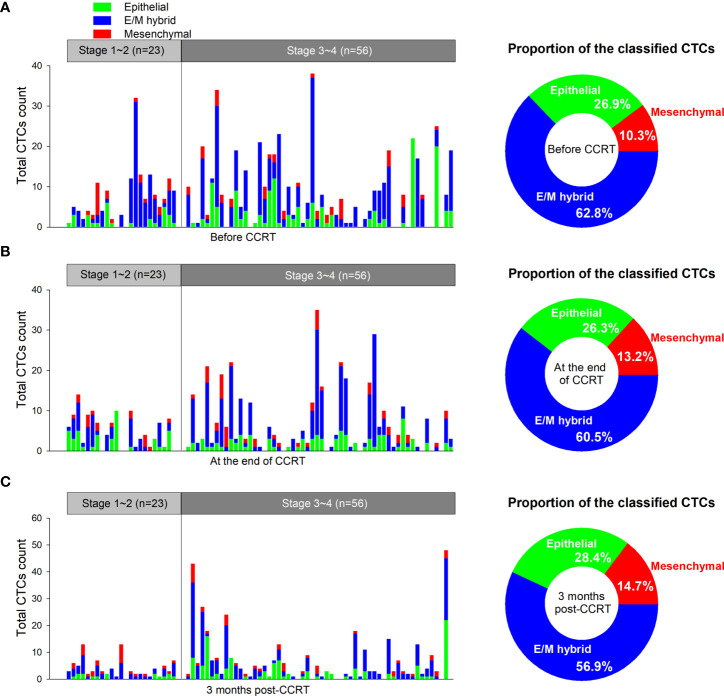
Numbers and distributions of circulating tumor cells (CTCs) before and after concurrent chemoradiotherapy (CCRT). **(A)** The numbers and distributions of the CTCs detected from each patient before CCRT are shown as tricolored bars. The proportions of phenotypically classified CTC subpopulations determined before CCRT are shown as a pie plot in the right panel. **(B)** Detection of CTCs at the end of CCRT. **(C)** Detection of CTCs at 3 months after CCRT. E/M, epithelial/mesenchymal.

Of the 92 patients enrolled in our study, 79 completed three CTC detections (before CCRT, at the end of CCRT, and 3 months after CCRT), which allowed us to assess the variations in CTC numbers and phenotypic distributions before and after treatment in a time-course manner. Among the 79 patients, 23 were diagnosed at an early stage (clinical stages I–II) and 56 were diagnosed at a progressive stage (clinical stages III–IV). A total of 662, 501, and 469 CTCs were detected at each of the three pre-designated time points in the 79 patients. The median total CTC counts were 7 (0–38), 4 (0–35), and 3 (0–48) before CCRT, at the end of CCRT, and 3 months after CCRT, respectively. Of the 662 CTCs detected before treatment, epithelial CTCs accounted for 26.9% (178/662), mesenchymal CTCs for 10.3% (68/662), and E/M hybrid CTCs for 63.8% (416/662). The numbers and distributions of CTCs detected from each patient and the total proportions of phenotypically classified CTCs at each designated time point are shown in **Figures 3A–C**.

**Figure 4:**
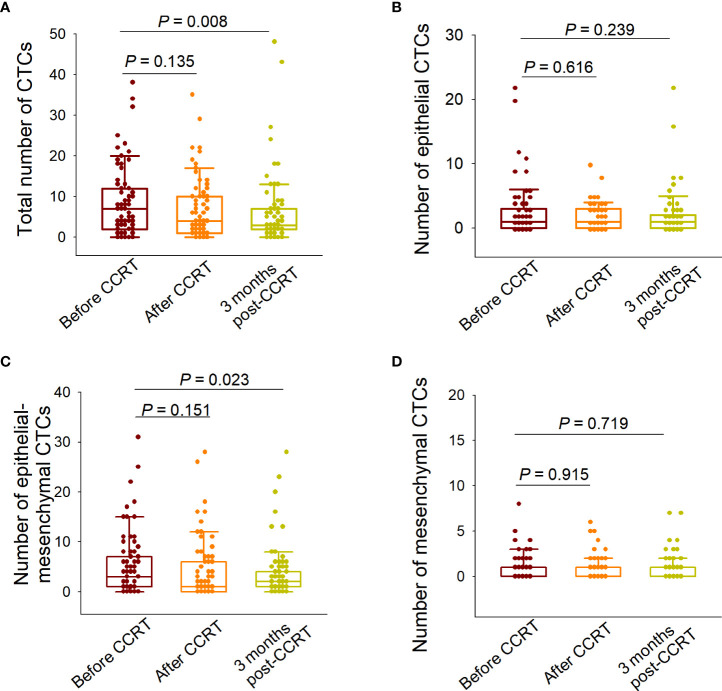
Variations in the numbers of total circulating tumor cells (CTCs) and each subpopulation before and after concurrent chemoradiotherapy (CCRT). **(A)** Comparison of the total CTC numbers detected before CCRT, at the end of CCRT, and 3 months after CCRT. **(B)** Comparison of the numbers of epithelial CTCs detected at the three predesignated time points. **(C)** Comparison of the numbers of epithelial/mesenchymal (E/M) hybrid CTCs detected at the three predesignated time points. **(D)** Comparison of the numbers of mesenchymal CTCs detected at the three predesignated time points. The paired-sample Wilcoxon test was performed for between-group comparisons of CTC numbers.

We first analyzed the changes in the total CTC numbers among the three predesignated time points. Although the total CTC numbers detected at the end of CCRT did not differ from those detected before treatment (*P*=0.135), they decreased significantly 3 months after CCRT (*P*=0.008) (**Figure 4A**). We then elucidated the changes in each phenotypically classified CTC sub-population. The epidermal or mesenchymal CTC numbers did not significantly change after treatment, both at the end of CCRT and 3 months after CCRT (**Figures 4B, D**). In addition, the numbers of E/M hybrid CTCs determined at the end of CCRT were not significantly different from those determined before treatment (*P*=0.151); however, the number of E/M hybrid CTCs detected at 3 months after CCRT was significantly decreased (*P*=0.023), showing a similar decreasing tendency to the number of total CTCs (**Figure 4C**).

### Prognostic value of the distribution of phenotypically classified CTCs

As the prognostic value of phenotypic classification of CTCs in predicting prognosis remains poorly understood, we compared the clinical outcomes of patients with NPC with different distributions of E/M hybrid CTCs. Of the 81 patients with detectable CTCs before treatment, 59 patients at a progressive stage (stage III–IV) who had a relatively high risk of locoregional or metastatic relapses after CCRT, the standard first-line treatment for NPC, were subjected to a follow-up study. According to the RNA ISH-based classification of CTCs, 34 and 25 patients were assigned to E/M hybrid-predominant (E/M hybrid CTCs >50%) and non-E/M hybrid-predominant (E/M hybrid CTCs ≤50%) groups, respectively. The baseline characteristics of the two groups are shown in **Table 1**.

**Table 1:** Baseline characteristics.

	E/M hybrid-predominant (n = 34)	Non-E/M hybrid- predominant (n = 25)	χ^2^	*P* value
Age (years)				
Median (range)	46 (17–63)	46 (29–71)		
≦45	15 (44.1%)	10 (40.0%)	0.100	0.752
>45	19 (55.9%)	15 (60.0%)		
Sex				
Male	30 (88.2%)	19 (76.0%)	0.786	0.375
Female	4 (11.8%)	6 (24.0%)		
Ethnicity				
Asian	34 (100%)	25 (100%)		
Histology				
Non-keratinizing squamous cell carcinoma	34 (100%)	25 (100%)		
T category				
T_1-2_	4 (11.8%)	8 (32.0%)	2.499	0.114
T_3-4_	30 (88.2%)	17 (68.0%)		
N category				
N_0-1_	21 (61.8%)	14 (56.0%)	0.198	0.656
N_2-3_	13 (38.2%)	11 (44.0%)		
Stage				
III	18 (52.9%)	18 (72.0%)	2.200	0.138
IV	16 (47.1%)	7 (28.0%)		
Pretreatment EBV DNA test				
Positive	17 (50.0%)	14 (56.0%)	0.208	0.648
Negative	17 (50.0%)	11 (44.0%)		

Data are presented as n (%) or median (range). χ^2^ was calculated by chi-square test with or without continuous calibration, or with Fisher’s exact test.

**Figure 5:**
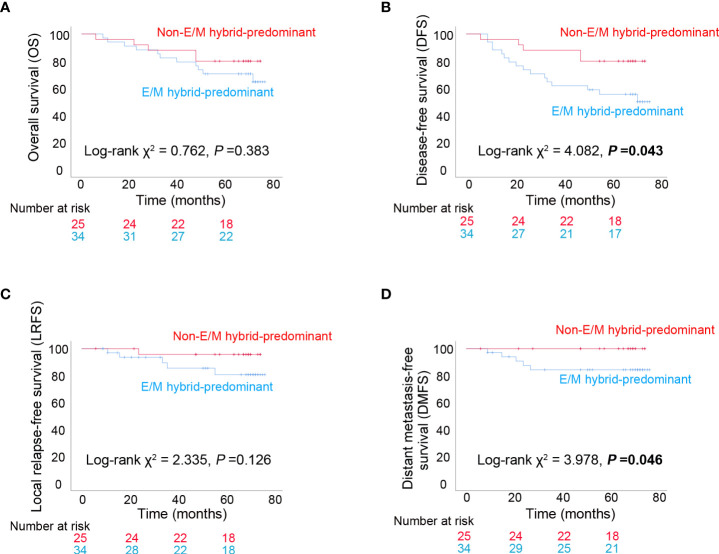
Long-term clinical outcomes among patients with progressive nasopharyngeal carcinoma. **(A)** Kaplan–Meier analysis of overall survival (OS) among patients with epithelial/mesenchymal (E/M) hybrid-predominant or non-E/M hybrid-predominant distribution of circulating tumor cells (CTCs). **(B)** Kaplan–Meier analysis of disease-free survival (DFS). **(C)** Kaplan–Meier analysis of local relapse-free survival (LRFS). **(D)** Kaplan–Meier analysis of distant metastasis-free survival (DMFS). The log-rank test was performed for between-group comparisons of survival curves.

The median follow-up period was 69 (6–77) months, with the following endpoint events: 16 patients died, 21 patients experienced disease progression, 6 patients experienced local recurrences, and 5 patients developed distant metastases. The 5-year OS rates were 70.6% (24/34) and 80% (20/25) in the E/M hybrid and non-E/M hybrid-predominant groups, respectively. The differences in OS (*P*=0.383) and LRFS (*P*=0.126) rates between the two groups were not statistically significant (**Figures 5A, C**). However, the DFS (*P*=0.043) and DMFS (*P*=0.046) rates were lower in the E/M hybrid-predominant group (**Figures 5B, D**), indicating that patients with progressive NPC with an E/M hybrid-predominant CTC distribution had unfavorable clinical outcomes.

## Discussion

The development of liquid biopsy technology has enabled the detection of CTCs in peripheral blood samples from patients with epithelial malignancies (7–10). Investigating the characteristics of CTCs has advanced our understanding of the underlying molecular pathways regulating blood-borne tumor dissemination at the single-cell level (9, 10, 25, 26). Interestingly, the capture of CTCs has enabled individualized testing of drug susceptibility through *ex vivo* culture of tumor cells directly derived from patients, establishing a paradigm of clinically valuable translation of liquid biopsy technology (27). Several alternative technological strategies have been established for CTC capture and isolation based on the presence or absence of tumor-specific epitopes, variations in physical properties such as size and density, and high-throughput image screening (7). In the present study, the CanPatrol™ CTC enrichment platform was used to enrich and isolate CTCs from NPC patients (22, 23). Owing to its highly efficient design, this system successfully isolated single CTCs and captured CTC clusters in our earlier study (12). The quality and integrity of the isolated CTCs were found to be adequate for nucleic acid extraction and subsequent whole-exome sequencing in a later study (19), indicating that the CanPatrol™ CTC enrichment system is suitable for CTC isolation and in-depth analyses. Furthermore, our previous whole-exome sequencing showed that various genes are frequently mutated in primary tumors and CTCs (19). Of these mutated genes, some are well-characterized functional oncogenes, such as *CYFIP2*, *NOP16*, and *ZNF117* (28–30), and genetic variations in others are closely associated with other cancers, such as *MOB3C* and *SSPO* (31, 32). It was also found that non-silent single nucleotide variations (non-silent SNVs) and insertion-deletion mutations were more frequent in CTCs than in primary tumor samples (19). Thus, our earlier work indicated that the CanPatrol CTC platform is a reliable system for isolating CTCs from NPC.

In our previous study, we preliminarily investigated the correlation between CTC numbers and clinical characteristics at baseline in patients with NPC and found that the CTC number was associated with clinical stage (12). However, no significant difference in total CTC numbers was observed among the T stages in the present study, which was supported by the finding that the total CTC number did not correlate with tumor size (**Figures 1A, D**). This inconsistency may be attributable to the different characteristics of the enrolled patients from different cohorts. In a study by He et al., CTCs were detected in 66.7% (22/33) of patients with NPC at all stages, but CTC numbers did not correlate with the tumor-node-metastasis (TNM) stage (13).

Zhang et al. reported that dynamic variations in CTC numbers can predict the efficacy of chemotherapy, and CTC karyotyping is associated with the sensitivity of NPC to chemotherapy (33). Wen et al. reported that CTCs determined in patients with NPC can serve as biomarkers for the dynamic assessment of therapeutic efficacy (34). In the present study, we compared the CTC numbers detected at three pre-designated time points (before CCRT, at the end of CCRT, and 3 months after CCRT) and found that the total CTC numbers were significantly reduced 3 months after CCRT (**Figure 4A**), which was consistent with our previous findings (12). Additionally, although the epithelial or mesenchymal CTC numbers did not significantly change at the end of CCRT or 3 months after CCRT, the E/M hybrid CTC numbers significantly decreased at 3 months after CCRT (**Figures 4B–D**). This indicates that the decline in total CTC numbers was mainly attributable to the reduced number of E/M hybrid CTCs. However, the significance of the variations in E/M hybrid CTC numbers for evaluating treatment responsiveness in patients with NPC who receive CCRT still needs to be further elucidated in a future study with a larger sample size according to the appropriate inclusion criteria.

In an earlier study, Sun et al. found that CTC numbers were significantly higher in metastatic patients with NPC than in non-metastatic patients (35). Ko et al. showed that serial analysis of CTCs was more sensitive to minimal residual disease in metastatic NPC than the conventional imaging approach (36). These findings strongly suggest the potential of CTC characterization for effectively predicting the prognosis of NPC. In contrast, the load of EBV DNA copies has been established as a valuable predictor of prognosis in NPC, as it is closely associated with the staging and risk of relapse and metastasis (37). He et al. reported that EBV-specific VCA-IgA levels in CTC-positive patients were higher than those in CTC-negative patients, and the CTC numbers were positively correlated with the EBV VCA-IgA level and EBV DNA load, suggesting a close association between CTC counts and EBV activation status (13). Ou et al. showed that the combination of CTC detection and EBV DNA detection was superior in predicting the prognosis of NPC compared to EBV DNA detection alone (38). You et al. further demonstrated that CTC numbers and EBV DNA levels determined before, after, and during first-line chemotherapy were clinically valuable in predicting prognosis in patients with metastatic NPC (39). In our study, the total CTC numbers did not differ between the EBV DNA-negative and -positive groups (**Figure 1C**). Interestingly, in our previous study, the levels of EBV-specific VCA-IgA and early antigen-IgA in E/M hybrid CTCs were significantly higher than those in epithelial or mesenchymal CTCs (12). More importantly, the EBV DNA level in E/M hybrid CTCs is higher than that in either epithelial or mesenchymal CTCs (12). Taken together, E/M hybrid CTCs, a subpopulation of CTCs with both epithelial and mesenchymal phenotypes, may be a preferable indicator of the tumor burden and can serve as a prognostic predictor in NPC. Therefore, we assessed the prognostic value of the distribution of the E/M hybrid CTCs. The enrolled patients were assigned to E/M hybrid-predominant and non-E/M hybrid-predominant groups. Our follow-up observations showed that the E/M hybrid-predominant distribution of CTCs predicted an unfavorable clinical outcome, with relatively lower DFS and DMFS rates observed among patients in the E/M hybrid-predominant group (**Figures 5B, D**). However, because the sample size of our study was relatively small, our findings need to be further verified in a prospective clinical trial with larger sample size.

Phenotypic classification of CTCs allows for a better understanding of the evolution of blood-borne spreading tumor cells, as the malignant characteristics of this tumor cell population have been demonstrated to differ from those of primary tumors and metastatic lesions (9, 26, 40). In this study, RNA ISH-based classification was introduced to distinguish the epithelial or mesenchymal phenotypes of CTCs using multicolor fluorescent probes (22, 23). The CanPatrol™ CTC enrichment platform enabled the assessment of both CTC numbers and the distributions of the CTC subpopulations in our study (**Figures 2A, B**). Tumor invasion and metastasis are highly dynamic processes driven by complex cellular signaling networks (41–43). The EMT of individual epithelial cancer cells is considered a critical initiating event in the invasion–metastasis cascade (35, 36). Epithelial-mesenchymal transition enables epithelial-derived cells within the primary tumor to acquire the ability to infiltrate the circulation (44, 45), whereas mesenchymal-to-epithelial transition allows disseminated cells to proliferate as epithelial metastatic deposits in remote sites (42, 43). Thus, the coexistence of epithelial and mesenchymal phenotypes may confer CTCs with the invasive properties required for the tumor invasion–metastasis cascade (41). Aceto et al. demonstrated that CTC clusters expressing plakoglobin, a well-known cell junction component, had a 23- to 50-fold increased metastatic potential compared to single CTCs (25). This suggests that maintaining epithelial characteristics is crucial for metastatic colonization and growth. We previously showed that the CTC cluster consists of E/M hybrid CTCs in NPC (12), which was demonstrated to have a high metastatic potential (25). CTCs with both epithelial and mesenchymal phenotypes may exhibit more aggressive characteristics than the epithelial or mesenchymal CTC subpopulations because of their highly invasive plasticity, which allows them to adapt to the evolving tumor metastatic microenvironment (41). However, of the 81 patients with detectable CTCs before treatment in the present study, sole epithelial CTCs were detected in 5 cases (5/81, 6.2%), sole E/M hybrid CTCs were detected in 18 cases (18/81, 22.2%), whereas no case was with sole mesenchymal CTCs (0/81, 0%). These results suggest that most of the enrolled cases were with more than one type of CTCs (58/81, 71.6%). Accordingly, the number of patients with a single type of CTCs was insufficient for prognosis evaluation. In addition, the correlation between the distribution of the classified CTCs and the expression patterns of EMT markers in primary NPC tissues remains unclear. This unresolved issue should be further elucidated by acquiring paired samples of primary tumors and CTCs in a subsequent study with sufficient sample size.

In the present study, the total CTC numbers decreased 3 months after CCRT, which was mainly attributable to the reduction in E/M hybrid CTC numbers along with the declining tumor burden. Patients with E/M hybrid-predominant distribution of CTCs had lower DFS and DMFS rates. The predominant distribution of E/M hybrid CTCs, a CTC subpopulation closely associated with EBV and endowed with high invasive plasticity, may serve as an effective predictor of the risk of remote metastasis in patients with progressive NPC.

## Data availability statement

The raw data supporting the conclusions of this article will be made available by the authors, without undue reservation.

## Ethics statement

The studies involving human participants were reviewed and approved by the ethics committee of the People’s Hospital of Guangxi Zhuang Autonomous Region. Written informed consent to participate in this study was provided by the participants’ legal guardian/next of kin.

## Author contributions

JZW and SQ conceived the study. ML, GL, XL, LY, YLW, FL, HO, YZW, WH, SX, and GD collected clinical data. JZW, WD, and JJW analyzed the data. JZW, WD and JJW prepared the manuscript. JZW and SQ supervised the study and revised the manuscript. All the authors have read and agreed to the submission and publication of this manuscript. All authors contributed to the article and approved the submitted version.

## Funding

This research was funded by the Guangxi Science and Technology Base and Talent Project (GuiKe-AD20297069), Guangxi Key Research and Development Program (GuiKe-AB18050011), the National Natural Science Foundation of China (82073004 and 81602390), and the Open Research Project of Key Laboratory of Early Prevention and Treatment for Regional High Frequency Tumor (Guangxi Medical University), Ministry of Education/Guangxi Key Laboratory of Early Prevention and Treatment for Regional High Frequency Tumor (GKE-KF202206).

## Acknowledgments

We would like to thank Editage (www.editage.cn) for English language editing.

## Conflict of interest

The authors declare that the research was conducted in the absence of any commercial or financial relationships that could be construed as potential conflicts of interest.

## Publisher’s note

All claims expressed in this article are solely those of the authors and do not necessarily represent those of their affiliated organizations, or those of the publisher, the editors and the reviewers. Any product that may be evaluated in this article, or claim that may be made by its manufacturer, is not guaranteed or endorsed by the publisher.
