# Supplementary figures and images for: Epithelial-mesenchymal transition classification of circulating tumor cells predicts clinical outcomes in progressive nasopharyngeal carcinoma
Source: Front Oncol. 2022 Sep 21;12:988458. doi: 10.3389/fonc.2022.988458 (PMC9532596; doi:10.3389/fonc.2022.988458)

# Study Design and Enrollment

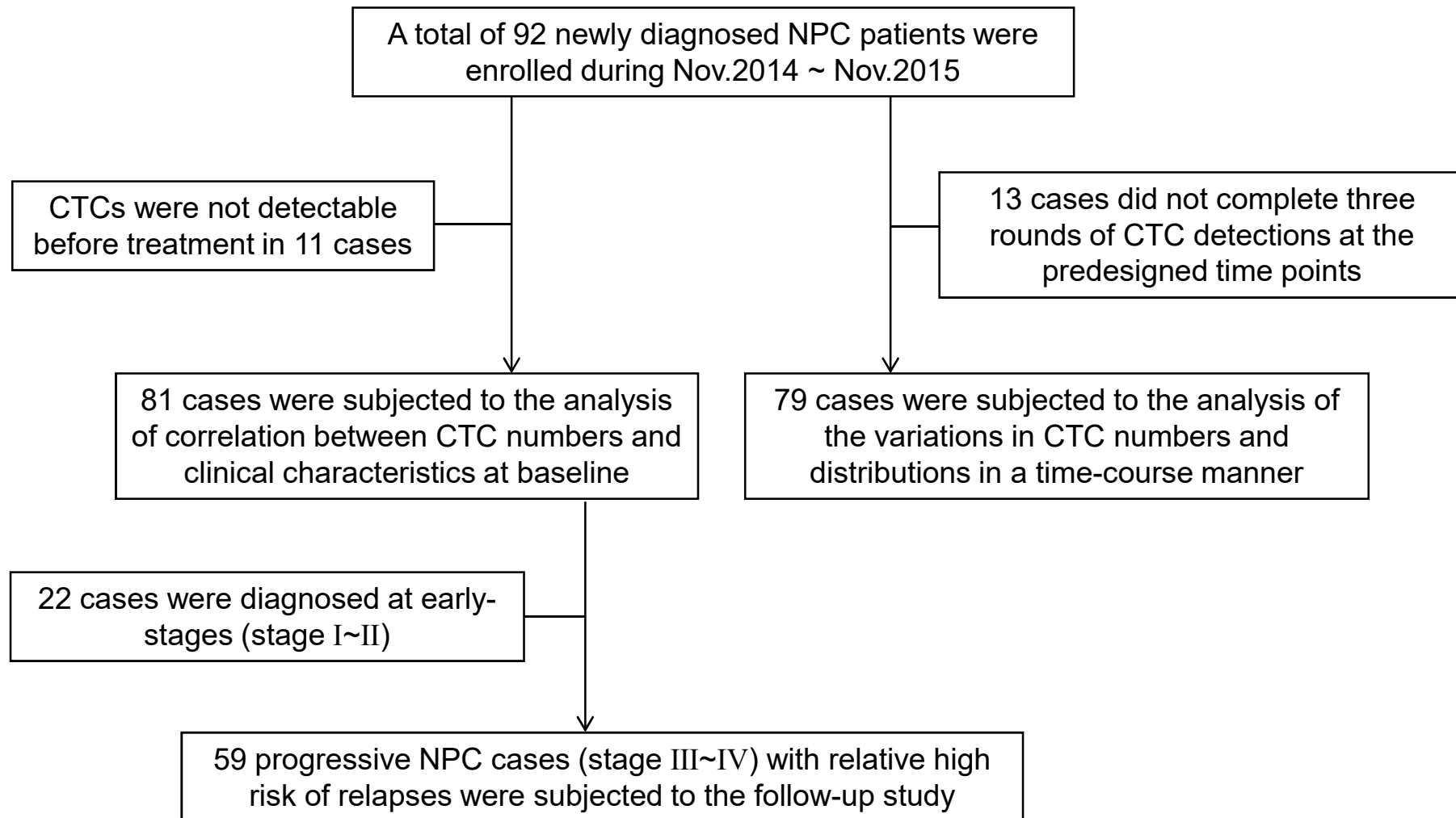

Supplement: Supplementary file 1 [file DataSheet_1.pdf]
